# Creolin in Dysentery, Serous Diarrhœa, and Summer Complaint

**Published:** 1891-10

**Authors:** Edward W. Watson

**Affiliations:** Philadelphia


### CREOLIN IN DYSENTERY, SEROUS DIARRHŒA, AND SUMMER COMPLAINT.

---

BY EDWARD W. WATSON, M. D., OF PHILADELPHIA.

---

Believing that many unrecorded bits of experience would be of use to the practitioner if published, I wish to call attention to the value of creolin as used by enema in dysentery and allied conditions.

The spring of 1890 in Philadelphia was marked by a considerable prevalence of acute dysentery of a typhoid character in scattered localities. It occurred to the writer while searching for a safe and efficient antiseptic to use creolin. It evidently occurred to others in the same way, for, after using it for some time successfully, I noticed in a current journal a quotation from a Russian source, wherein its use was advocated for the same complaint. In regard to personal experience, the small epidemic enabled me to employ it during the month of June, 1890, on twenty-three cases. These were of all grades. Some at the beginning, others almost exhausted; but in all but one the use of a large enema, containing one-half of one per cent. of creolin,—one drachm to a pint,—was followed by results quite surprising. One of the worst cases, a girl of 12, in the fourth day had had between thirty and forty stools, bloody, shreddy, and fetid, and, after an enema at 7 p. m., did not require the pan, had no stool or attempt, slept quietly all night, and had a formed movement in the morning. This, as in all the cases, without any other medication. Another, a boy of ten, seemed moribund, but after an enema, the number of mucous stools, which had been in the previous twelve hours thirty-five or forty, was reduced to one, and after a subsequent enema normal passages occurred. This child's two brothers were attacked during my second visit with violent symptoms. An enema was administered to each, and no subsequent sign of the disease occurred at all. In short, all the cases recovered, in whatever stage they were, from the commencement of the treatment. Contrasting this with the usual course of dysentery, and its exasperating uncertainty, even with the saline treatment, one can claim that so far creolin is without an equal. The one exception puzzled me for some time, until I discovered that the mother, ignorant of how to use an enema, had simply omitted it, and lied.

But from this use of the creolin to another was but a step. As the season advanced numerous cases of "summer complaint"—colitis and entero-colitis—occurred as usual, and it occurred to the writer to try the same method. These were infants, and the amount of creolin was reduced to one-half drachm to a pint of warm water, and twice a day the bowels were flushed with a gravity syringe. Here the result was also everything that could be expected. Bearing in mind that it was not employed in the cases of *sudden* onset, bounding and rapid diarrhœa, which are called cholera infantum, but in the more frequent, equally dangerous, but more protracted cases, where diarrhœa sets in, keeps up, and gradually wears out the infant, with recurring exacerbations, till finally often the so-called cholera infantum extends with such violence as to destroy what little resistance and life remain. In short, wherever there was diarrhœa, with green and bloody or mucous stools, undigested curds, etc., the remedy, when faithfully used, rapidly restored the natural character and frequency to the evacuation, and the immense improvement to the child showed that besides remedying the catarrhal process, there must be also something to be credited to the washing out of deleterious secretions.

Having little or no dysentery to treat up to this date, in 1891, I have been employing the remedy in these cases this year with the same success. I would also note that during the winter, occassionally in young infants, I met with cases of sudden and rapidly-exhausting serious diarrhœa. All of these, which were treated in this way, three in all, were so treated after the usual medication had failed, and with rapid success, and I think that there is enough in it to warrant calling attention to it and the giving it a further trial.—*Therapeutic Gazette,* Aug. 15th, 1891.

